# Closed-loop regulation of arterial pressure after acute brain death

**DOI:** 10.1007/s10877-017-0033-z

**Published:** 2017-06-10

**Authors:** Kristian Soltesz, Trygve Sjöberg, Tomas Jansson, Rolf Johansson, Anders Robertsson, Audrius Paskevicius, Quiming Liao, Guangqi Qin, Stig Steen

**Affiliations:** 1 Department Automatic Control, Lund University, P.O. Box 118, SE-221 00 Lund, Sweden; 2 Department Cardiothoracic Surgery, Lund University and Skåne University Hopital, Lund, Sweden; 3 Department Clinical Sciences Lund, Biomedical Engineering, Lund University, Lund, Sweden and Medical Services, Skåne University Hospital, Lund, Sweden

**Keywords:** Hemodynamics, Blood pressure, Closed-loop control, Brain death

## Abstract

The purpose of this concept study was to investigate the possibility of automatic mean arterial pressure (MAP) regulation in a porcine heart-beating brain death (BD) model. Hemodynamic stability of BD donors is necessary for maintaining acceptable quality of donated organs for transplantation. Manual stabilization is challenging, due to the lack of vasomotor function in BD donors. Closed-loop stabilization therefore has the potential of increasing availability of acceptable donor organs, and serves to indicate feasibility within less demanding patient groups. A dynamic model of nitroglycerine pharmacology, suitable for controller synthesis, was identified from an experiment involving an anesthetized pig, using a gradient-based output error method. The model was used to synthesize a robust PID controller for hypertension prevention, evaluated in a second experiment, on a second, brain dead, pig. Hypotension was simultaneously prevented using closed-loop controlled infusion of noradrenaline, by means of a previously published controller. A linear model of low order, with variable (uncertain) gain, was sufficient to describe the dynamics to be controlled. The robustly tuned PID controller utilized in the second experiment kept the MAP within a user-defined range. The system was able to prevent hypertension, exceeding a reference of 100 mmHg by more than 10%, during 98% of a 12 h experiment. This early work demonstrates feasibility of the investigated modelling and control synthesis approach, for the purpose of maintaining normotension in a porcine BD model. There remains a need to characterize individual variability, in order to ensure robust performance over the expected population.

## Introduction

### Pathophysiology and treatment of heart-beating brain dead donors

Heart-beating brain dead (BD) donors are the largest source of donor organs [1]. Upon acute BD, homeostasis is lost, leading to the rapid degradation of body organs. The incidence of BD is associated with a dramatic increase in plasma catecholamine levels. This effect, resulting in a brief (<30 min) episode of hypertension is referred to as the catecholamine storm [2]. As catecholamines are eliminated from the blood stream, blood vessels lose their tone, resulting in severe hypotension. In the absence of treatment, the body experiences systemic circulatory collapse, leading to a loss of adequate organ perfusion. Relatedly, plasma concentration of cortisol, antiduretic hormone (vasopressin), and thyroid hormones decrease upon BD [2].

In order to compensate for the mentioned changes, BD donors are subject to hormone therapy. Internationally recognized guidelines for treatment of BD donors are provided in [3, 4]. This study is based on local (Swedish) recommendations [5], derived from the aforementioned guidelines. Prevention of hypotension is the most challenging of the treatment goals. The guidelines dictate maintenance of a mean arterial pressure (MAP) exceeding 60–70 mmHg, while remaining below 100 mmHg. This can be partially achieved by intravenous infusion of noradrenaline. However, infusion rates exceeding 0.05 μg/min/(kg body weight), are not feasible due to elevated risk of cardiac graft dysfunction, and higher early and late recipient mortality. Consequently, hormonal therapy is complemented by forced fluid therapy, with the adverse effect of drastically increasing the risk of lung edema [6]. It has been demonstrated in a porcine BD model, that a sufficient MAP can be maintained throughout a 24 h treatment window,^1^ in the absence of forced fluid therapy, by blocking neural catecholamine reuptake [7].

While previous research [7, 8] has focused on maintaining a sufficient MAP, clinical care of BD donors would additionally benefit from the capability to avoid periods of excessive hypertension. Most notably, the catecholamine storm associated with BD, results in decreased tissue perfusion due to drastically increased systemic (SVR) and pulmonary (PVR) vascular resistance [2]. It has been observed in a primate BD model, that the subsequent fall of PVR is more abrupt than that of SVR, resulting in blood pooling in the lungs, and consequently an increased risk of pulmonary edema [9].

A further motivation for this work is that sustained episodes of elevated arterial pressure, exceeding the recommended upper limit of MAP >100 mmHg, occurred spontaneously after 5–10 h in the utilized porcine BD model, during all experiments underlying [10]. These episodes could not be explained by the combined administration of catecholamines and reuptake blocker, as they did not cease upon halted administration of the mentioned drugs.

### Closed-loop hemodynamic management

While the human body is rich in closed-loop control systems (vasomotor and blood glucose regulation, to mention two), synthetic counterparts are still rare in clinical medicine. Studies aimed at closed-loop control of anesthetic depth and analgesia by means of EEG-guided infusion of anesthetic and analgesic drugs have proven successful [11, 12]. The purpose of such systems is to alleviate clinicians of repetitive low-level tasks, such as intermittent infusion rate adjustments.

Following successful studies on closed-loop anesthesia systems, the prospect of closed-loop controlled hemodynamic stabilization, including the control of intravascular volume, has been proposed as a challenging interdisciplinary research problem [12, 13]. Relatedly, potential clinical benefit of closed-loop controlled blood pressure management was highlighted in a recent in silico simulation study [14]. A comprehensive review of the current state of the art regarding closed-loop controlled hemodynamic management in humans is provided in [15]. The review reveals that apart from efforts in hypertension managements using sodium nitroprusside in the 1980s and early 1990s, there exist very few reports directly related to the work presented herein.

The thesis underlying the work presented herein, is that relatively simple system identification and robust control methods are sufficient to control MAP in a porcine BD model. The use of a porcine model is motivated by the fact that large animal models are the closest available approximation of human cardiovascular physiology, see e.g. [16].

**Fig. 1:**
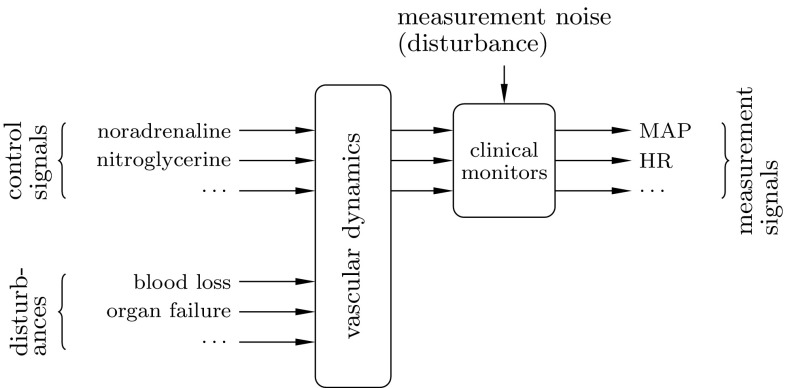
The BD organ donor, illustrated from a control system perspective

**Fig. 2:**
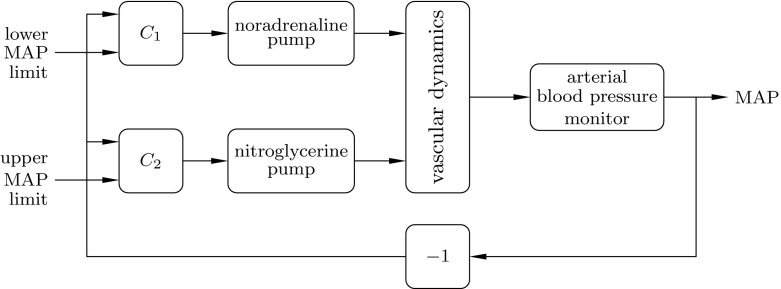
Control system configuration, used during the close-loop controlled experiment. The controller $C_1$ administers noradrenaline to prevent hypotension, while $C_2$ administers nitroglycerine to prevent hypertension. Remaining drugs of Table 1 were not under closed-loop control

To investigate the thesis, a control system with two loops, schematically depicted in Fig. 2 was constructed. In this context, the process to be controlled are the vascular dynamics, conceptually illustrated by Fig. 1. One of the loops prevents hypotension by means of noradrenaline infusion, as described in [10]. A second controller, being the focus of this paper, is simultaneously acting to prevent hypertension, by means of nitroglycerine infusion. Nitroglycerine is a potent vasodilator, suited for the task, as it lacks side effect, which would limit its administration to BD donors [17].

## Methods

### Experimental setup

**Fig. 3:**
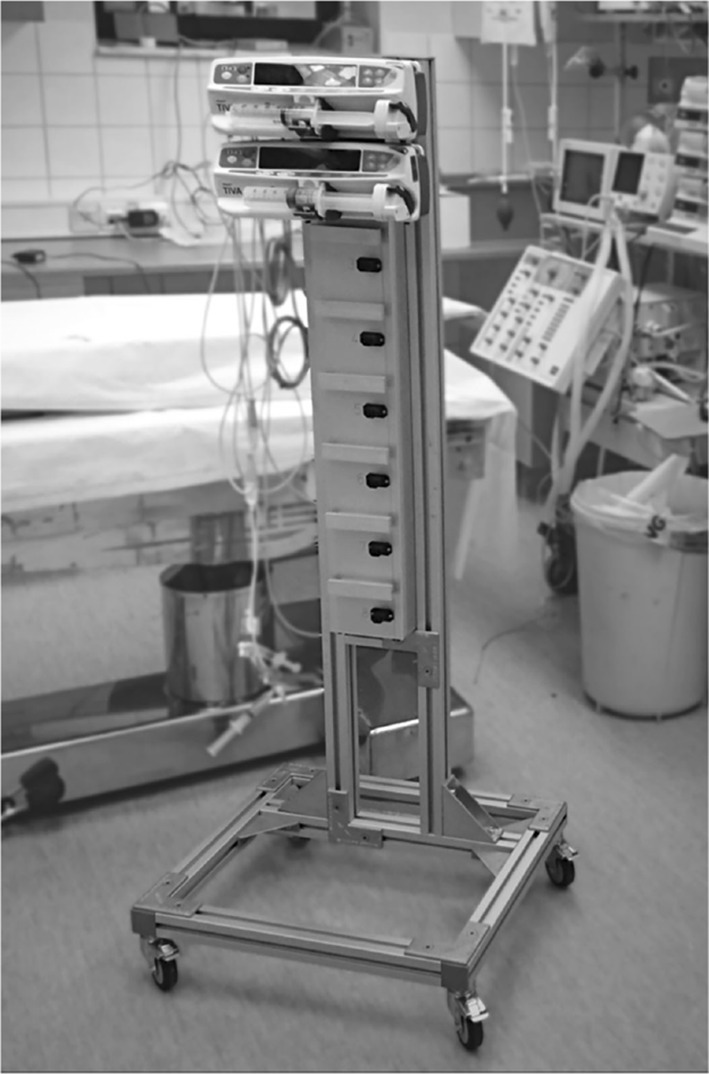
Prototype closed-loop hemodynamic stabilization system, with two (out of eight possible) computer controlled infusion pumps attached. Connectors for blood pressure transducers and user interface PC are situated on the backside of the column

A modular control system, enabling simultaneous computer control of eight infusion pumps (Carefusion Alaris TIVA; BD, Franklin Lakes, NJ) was constructed. It is shown in Fig. 3, with two infusion pumps attached. In addition to the pumps, the system has connection ports for invasive blood pressure transducers (DTXPlus; Argon Medical, Plano, TX). Signal processing, control and GUI are implemented on a PC, communicating with the pumps and sensors via RS232 over a USB cable.

For the experiments described herein, the system was fitted with five infusion pumps, according to Table 1. Arterial pressure measurements were collected at 100 Hz, and used to compute mean arterial pressure (MAP), as well as diastolic and systolic pressure. All measurement and control signals were displayed on the graphical user interface, and logged at 1 Hz.

**Table 1:** Drugs per syringe pump

Pump	Drug	Conc. (mg/ml)
1	Noradrenaline	0.02
2	Cocaine	0.02
3	Nitroglycerine	5.0
4	Adrenaline	0.02
5	Desmopressin	0.72

Prevention of hypotension by means of closed-loop co-administration of noradrenaline and cocaine was studied in [10]. This work focused on simultaneously preventing hypertension through closed-loop controlled infusion of nitroglycerine

### Animal preparation

Two experiments, each involving one 40 kg Swedish native breed pig, were conducted, as subsequently described. The animals were subject to anesthesia, induced through intramuscular injection of atropine (0.5 mg) (Atropine; Mylan AB, Stockholm, Sweden), xylazine (100 mg) (Rompun Vet; Bayer A.B., Solna, Sweden), and ketamine (800 mg) (Ketaminol Vet.; Intervet, Boxmeer, Netherlands). Fentanyl (160 μg) (Fentanyl B.; Braun, Melsungen, Germany) and midazolam (16 mg) (Midazolam Panpharma; Panpharma S.A., Trittau, Germany) were given intravenously before tracheostomy. Anesthesia and muscle relaxation were maintained with continuous intravenous infusion of a mixture of ketamine (10 mg/kg/h) and rocoronium (1.5 mg/kg/h) (Fresenius Kabi, Uppsala, Sweden).

The animals were placed in supine position, and mechanically ventilated (Servo Ventilator 300; Siemens AB, Solna, Sweden), using volume-controlled and pressure regulated ventilation, with a minute volume of 100–150 ml/(kg body weight) at 20 breaths per minute. Positive end-expiratory pressure (PEEP) was adjusted to 5 cm H_2_O, inspired oxygen fraction was 0.5, and end tidal CO_2_ was kept between 4.5 and 5.5 kPa through the minute volume regulation.

A suprapubic cysostomy was performed to catheterize the urine bladder. A urimeter was subsequently connected via a Foley catheter, in order to monitor urine production. A saline solution (9 mg/ml) (Sodium chloride; Baxter Medical AB, Kista, Sweden) was continuously infused (3 ml/kg/h) to compensate for fluid losses due to urination, respiration and perspiration, as well as bleeding. Heating of the table was manually adjusted to maintain a body core temperature of 36–38 °C.

Subsequently, the animals were connected to the control system; lines from the syringe pumps were connected to the line carrying Ringer-acetate (Baxter Viaflo; Baxter Medical AB, Kista, Sweden), and catheters for continuous blood pressure measurement were inserted in the ascending aorta and superior vena cava, respectively, and connected to the system shown in Fig. 3. The pressure transducers were continuously rinsed with a Ringer-heparin solution (5 IU/ml) (Heparin LEO; Vianex Factory A, Athens, Greece) to prevent coagulation on the substrate. Arterial blood gas samples were drawn and analyzed intermittently (ABL 700; Radiometer, Copenhagen, Denmark), to ensure normal values.

Following the preparation phase, the two experiments differed, as explained below, under the “System identification” and “Closed-loop demonstration” sections, respectively. In both experiments, total BD was induced by surgical decapitation between C_2_ and C_3_, as explained in [7].

###  System identification

The purpose of the first experiment was to obtain a dynamical model relating nitroglycerine infusion rate to MAP. In both porcines and humans, nitroglycerine has very short time-to-peak effect, and half-life (both on a sub-minute or minute time scale). While this makes manual titration challenging, it becomes an advantage in the closed-loop control framework—it allows the controller to react faster to both disturbances and reference changes, as it does not have to ‘wait' for slow drug dynamics.

**Fig. 4:**
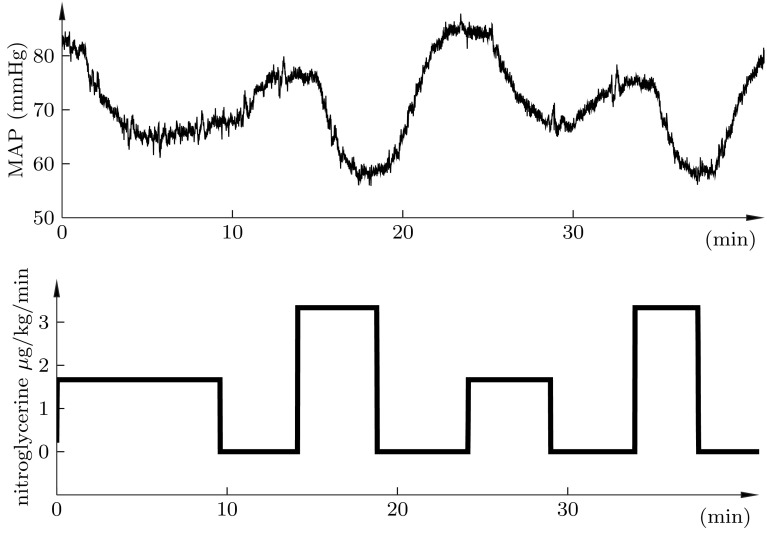
Upper plot shows time evolution of MAP, resulting from the infusion profile of the lower plot. Step sizes correspond to infusion rate changes of 5 ml/h, and 10 ml/h, respectively. MAP was stable at the start time of the experiment

The identification experiment conducted, comprised a sequence of alternating positive and negative step changes of nitroglycerine infusion, as shown in Fig. 4.

Pharmacokinetics and dynamics are traditionally described by compartment models. They constitute a subclass of linear time-invariant systems, which can be defined by *transfer functions* in the frequency domain. For our purposes (closed-loop control), the physiological interpretation of individual compartments is not of explicit interest. Therefore, it may suffice to assert a low-order model structure.

1 $$\begin{aligned} P(s) = \dfrac{K}{(sT_1+1)(sT_2 +1)}e^{-sL}, \end{aligned}$$

where *K* ($\varDelta$mmHg/$\varDelta$ml/h) denotes the static gain, $T_1$ (s), and $T_2$ (s) are time constants explaining the temporal evolution of the response, and *L* (s) is a pure time delay between change of infusion rate and noticeable effect in the MAP.

**Fig. 5:**
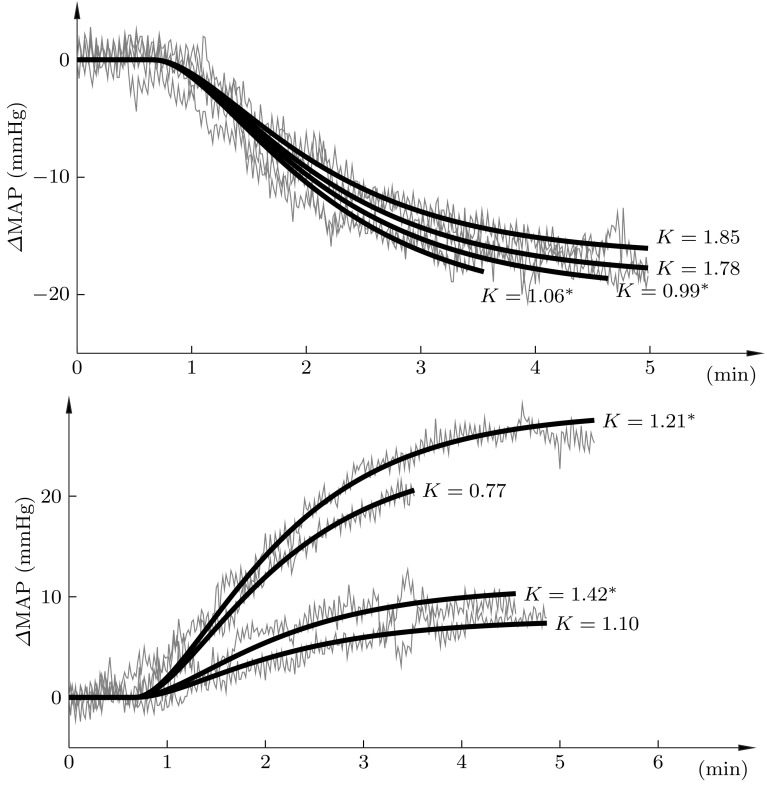
Superimposition of the positive (*upper*) and negative (*lower*) step responses of Fig. 4 (*grey*), together with corresponding responses of the identified models (*black*). The static gain of each model is shown explicitly in the figure. Values marked * Asterisk* correspond to the larger of the two step sizes of Fig. 4

A superimposition of the positive and negative changes in MAP are shown in Fig. 5, in grey, together with corresponding responses of identified second-order time-delayed transfer functions of the form (1), in black. While the identified gain parameter *K* varied between the responses; $K\in \left\{ 0.77,0.98,1.06,1.10,1.21,1.42, 1.68,1.78\right\},$ the same dynamic parameters; $T_1=30,$ $T_2=70,$ and $L=40,$ were able to accurately describe the dynamics, as shown in Fig. 5.

### Closed-loop demonstration

With a set of dynamic models (one model per *K*-value) explaining the relation between changes in nitroglycerine infusion rate and MAP, it was possible to synthesize a closed-loop controller. Owing to its simplicity and reliability, a PID controller,

2 $$\begin{aligned} C(s)=k_p+k_i s + \frac{k_d}{s}, \end{aligned}$$

was chosen, where, $k_p$ (ml/s/mmHg), $k_i$ (ml/mmHg) and $k_d$ (ml/s/mmHg/s) are constant parameters.

In order to attenuate high-frequency measurement noise, the controller was connected in series with a second-order low-pass filter with transfer function ${(sT_f+1)^{-2}},$ where $T_f=8$ s was chosen to facilitate a trade-off between noise attenuation and controller performance.

Tuning of the PID controller for the identified model set was performed using an optimization-based algorithm, as explained in detail in [10]. Both a PID controller ($k_p=-0.41,$ $k_i=-6.64 \times 10^{-3},$ $k_d=-20.3,$) and a PI controller ($k_p=-0.21,$ $k_i=\,-2.7\times 10^{-3}$, $k_d=0$) were obtained. The PID controller resulted in superior disturbance rejection properties (load step integrated absolute error) for the model set, and was hence chosen in favor of its PI counterpart.

The controller and filter were discretized and implemented in the control system of Fig. 3. Clamping integrator anti-windup was added, as described in [18]. The system was connected to the second pig prior to decapitation. Nitroglycerine was administered under closed-loop control, with the controller described above, and an (upper) MAP reference of 100 mmHg, to prevent hypertension.

Noradrenaline was simultaneously administered under closed-loop control, with a (lower) MAP reference of 65 mmHg, as described in [10]. Cocaine was infused at a fixed ratio (1 mg/mg) to noradrenaline. Desmopressin administration was manually adjusted to maintain normal urine production [5].

The 24 h treatment window was divided into two episodes of 12 h each. The first of these is more challenging from a control perspective due to the catecholamine storm, and associated hypertension, with subsequent hypotension and circulatory collapse in absence of therapy. Consequently, closed-loop controlled drug administration was enabled during the first 12 h episode. Nitroglycerine infusion was ceased during the second 12 h episode, in order to evaluate the effectiveness of the closed-loop controlled drug administration.

## Results

**Fig. 6:**
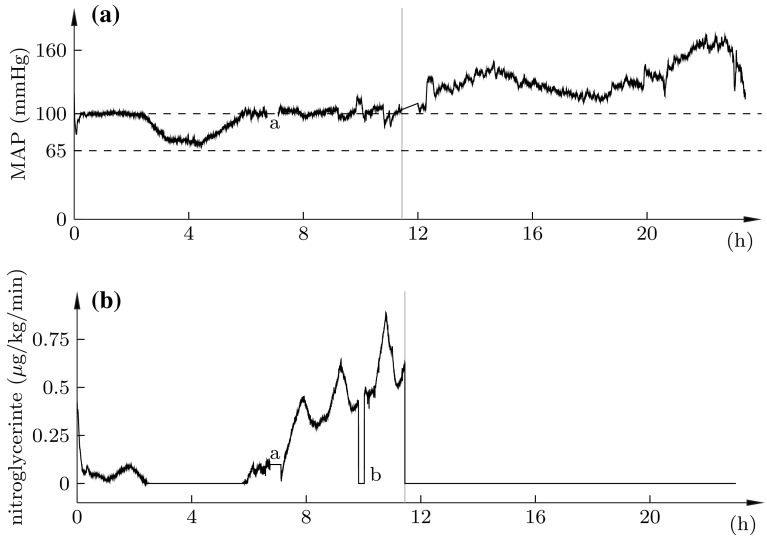
Nitroglycerine infusion (*bottom*) and resulting MAP (*top*, *black*). *Vertical grey line* shows division between closed-loop control (*left*), and halted nitroglycerine infusion (*right*). *Dashed lines* indicate limits of the desired MAP range [5].* Marks* show: **a** missing data due replacement of arterial pressure transducer; **b** infusion pause due to syringe replacement

The outcome of the full 24 h experiment is shown in Fig. 6. The vertical grey line indicated the division between the first 12 h of closed-loop control, and the subsequent 12 h of halted nitroglycerine infusion. The top plot shows the MAP (solid), and the recommended [5] MAP limits (dashed). The bottom plot shows the corresponding nitroglycerine infusion profile.

**Fig. 7:**
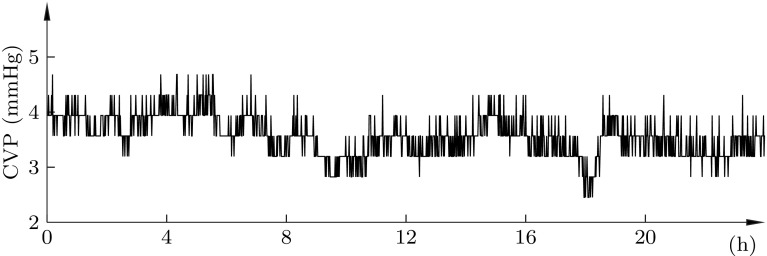
Central venous pressure throughout the 24 h experiment

The corresponding central venous pressure, not subject to closed-loop control, is shown in Fig. 7. It remained ≤10 mmHg, as per the recommendation [5], throughout the experiment.

Decapitation was performed at t = 0. Owing to the responsiveness of the closed-loop nitroglycerine controller, the elevation of arterial pressure associated with the catecholamine storm was almost completely eliminated. This is to be compared with the response in absence of the controller, where previous publications reported MAP elevations to 170 mmHg [7], and 200 mmHg [10], respectively.

The MAP fell below 100 mmHg at t = 2 h 23 min, resulting in the closed-loop controller ceasing the administration of nitroglycerine. Unlike in previously conducted experiments [7, 10], the MAP did subsequently not fall below 65 mmHg. Consequently, neither noradrenaline nor cocaine were administered by the system throughout the experiment.

The increased nitroglycerine demand, starting at $t=$ 5 h 47 min of the first episode, indicates that MAP would have exceeded 100 mmHg, in absence of closed-loop controlled nitroglycerine. Further support for this are the witnessed MAP increases during the time of syringe change, marked* a* in Fig. 6, and throughout the second 12 h episode (grey), following termination of closed-loop controlled drug administration.

**Fig. 8:**
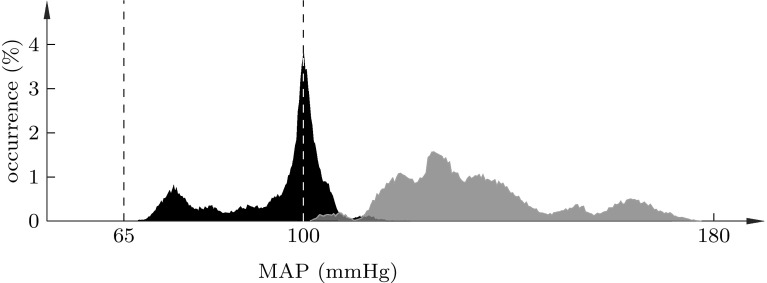
MAP distribution during the 12 h episodes with (*black*) and without (*grey*) closed-loop control. *Dashed lines* indicate the recommended [5] 65–100 mmHg range

The MAP stayed within the recommended range of 65–100 mmHg 61% of the time, and in the range 65–110 mmHg 98% of the time, during the 12 h of closed-loop control, and 1% of the time during the subsequent 12 h in absence of closed-loop control. A histogram showing the distribution of time-in-range is shown in Fig. 8. The average nitroglycerine infusion rate during the 12 h episode under closed-loop control was 0.17 g/kg/min.

**Fig. 9:**
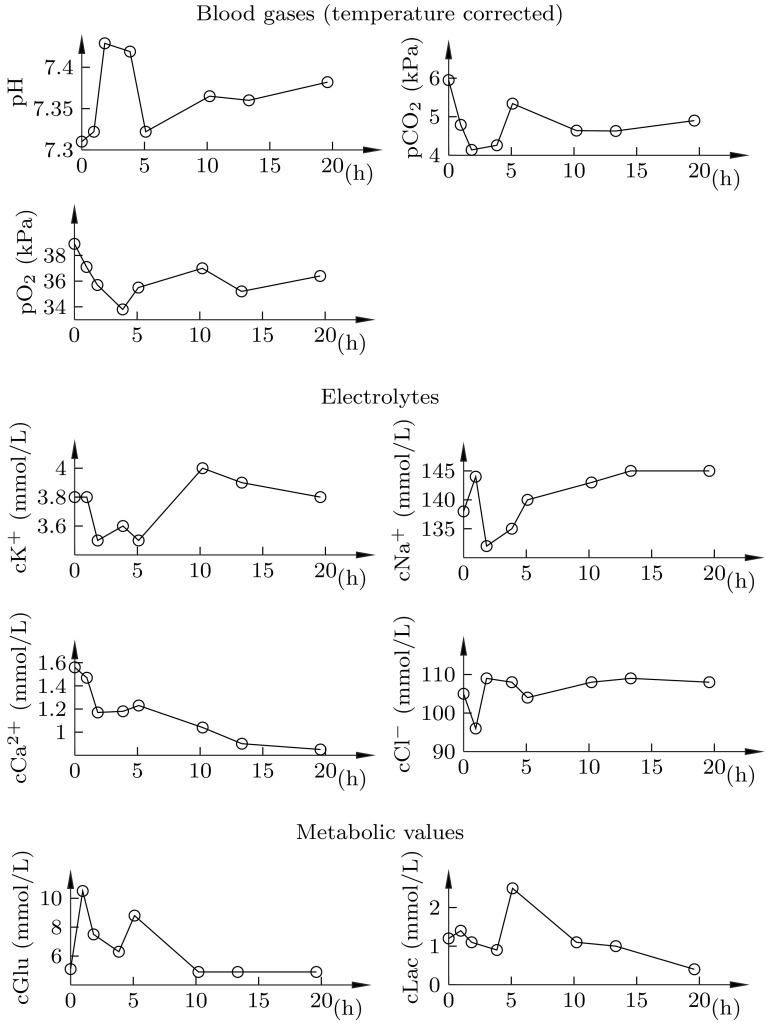
Arterial blood gas analysis results. *Circles* indicate sampling instances

As seen in Fig. 9, normal blood gas values were maintained throughout the experiment.

## Discussion

The results support the thesis that simple modelling and control techniques are adequate maintaining normotension in the studied porcine BD model. The sufficiency of low-order linear models is supported by the fits shown in Fig. 5, and indirectly by the control performance of Fig. 6, which additionally indicates sufficiency of using a PID controller.

The static gain variations seen for both nitroglycerine (Fig. 4) and noradrenaline [10] response models has been met by tuning the controller in a robust, or conservative, fashion, at the cost of degraded performance (i.e., error responsiveness). An alternative to this paradigm is *adaptation*, where the static gain parameter is estimated online throughout the experiment, and the controller parameters updated accordingly.

Another finding is the spontaneous arterial pressure increase, witnessed in the experiments underlying this and previous [10] work. Whether this phenomenon is specific to our porcine BD model, should be investigated. Closed-loop control of a vasodilator, such as nitroglycerine, is motivated regardless, as it can eliminate the potentially harmful episode of severe hypertension, associated with the catecholamine storm.

As with concept studies, this work is limited by sample size. While the utilized controller tuning technique is capable of handling model sets as well as models with unstructured uncertainty [19], it remains to characterize the underlying individual variability. A related question is exactly how well (individual and population) porcine models translate to human physiology.

## Footnotes

1 The authors are based in Sweden, where treatment of BD donors is limited to 24 h by national regulations.
